# Comparison of the effects of remifentanil-based general anesthesia and popliteal nerve block on postoperative pain and hemodynamic stability in diabetic patients undergoing distal foot amputation: A retrospective observational study

**DOI:** 10.1097/MD.0000000000004302

**Published:** 2016-07-22

**Authors:** Na Young Kim, Ki-Young Lee, Sun Joon Bai, Jung Hwa Hong, Jinwoo Lee, Jong Min Park, Shin Hyung Kim

**Affiliations:** a Department of Anesthesiology and Pain Medicine; b Anesthesia and Pain Research Institute; c Department of Research Affairs, Biostatistics Collaboration Units; d Department of Orthopedic Surgery, Yonsei University College of Medicine, Seoul, Republic of Korea.

**Keywords:** diabetic foot, distal foot amputation, hemodynamic stability, popliteal nerve block, postoperative complication, postoperative pain, remifentanil-based general anesthesia

## Abstract

Diabetic foot ulcer is the most common cause of diabetes-associated nontraumatic lower extremity amputation. Most patients who undergo lower extremity amputation for a diabetic foot have had diabetes for a long time and suffer from multiorgan disorder; thus, it can be a challenge to ensure sufficient anesthetic and analgesic effects while maintaining stable hemodynamics. Recently, peripheral nerve block has gained popularity owing to its attenuating effects of systemic concerns. This retrospective observational study aimed to compare the effects of remifentanil-based general anesthesia (GEA) and popliteal nerve block (PNB) on postoperative pain and hemodynamic stability in diabetic patients undergoing distal foot amputation.

A total of 59 consecutive patients with a diabetic foot who underwent distal foot amputation between January 2012 and May 2014 were retrospectively reviewed. Patients received remifentanil-based GEA (GEA group, n = 32) or PNB (PNB group, n = 27). The primary outcomes were to evaluate postoperative analgesic effects and perioperative hemodynamics. Also, postoperative pulmonary complications and 6-month mortality were assessed as secondary outcomes.

Significant differences in pain scores using numeric rating scale were observed between the groups in a linear mixed model analysis (*P*_Group×Time_ = 0.044). Even after post hoc analysis with the Bonferroni correction, the numeric rating scale scores were significantly lower in the PNB group. Furthermore, patients in the PNB group required less pethidine during the first 6 hours after surgery (27 ± 28 vs 9 ± 18 mg; *P* = 0.013). The GEA group had a lower mean blood pressure (Bonferroni-corrected *P* < 0.01), despite receiving more ephedrine (*P* < 0.001). Significantly more patients in the GEA group suffered from postoperative pneumonia and required the management in intensive care unit (*P* = 0.030 and 0.038, respectively). However, the groups did not differ in terms of 6-month mortality.

This study demonstrated that compared with remifentanil-based GEA, PNB might be a favorable option for diabetic patients undergoing distal foot amputation, despite the lack of significant mortality benefits, as PNB was associated with improved postoperative analgesia, hemodynamic stability, and a low incidence of pulmonary complications during the immediate postoperative period, especially in high-risk patients.

## 1 Introduction

Diabetes mellitus (DM) is one of the most common metabolic disorders and is increasing in prevalence.^[1,2]^ The risk of lower extremity diseases such as peripheral arterial disease, peripheral neuropathy, and foot ulceration is higher among diabetic patients than among nondiabetic patients, and nearly 10% to 25% of diabetic patients could develop a foot ulcer during their lifetime.^[3–5]^ Surgical interventions may be needed to manage diabetic foot infections; these vary from minor to major interventions, such as debridement or amputation.^[6,7]^ The rate of lower extremity amputation (LEA) among patients with diabetes may be as high as 70%, and the most frequent cause of nontraumatic LEA is a diabetic foot ulcer.^[8–10]^

The importance of optimal postoperative pain control has been well recognized for improving postoperative recovery and outcome, which may also help attenuate the possibility of developing chronic pain.^[11,12]^ Particularly in high-risk patients, the value of pain management for controlling postoperative complications has been clearly reported.^[13]^ Meanwhile, most patients who undergo LEA for a diabetic foot have had diabetes for a long time, are elderly, have at least 1 comorbid disease, and suffer from multiorgan disorder.^[14,15]^ In addition, approximately 30% to 40% of the patients with a diabetic foot have undergone at least 1 reamputation after a previous debridement or minor amputation.^[9]^ Therefore, it can be a challenge to ensure sufficient anesthetic and analgesic effects while maintaining stable hemodynamics in these patients.

Recently, peripheral nerve block, which is performed under ultrasound guidance and local anesthesia, has become popular because of its effects, which attenuate systemic concerns that might result from general anesthesia (GEA) and its adverse effects on cardiopulmonary functions.^[16]^ Given the increasing interest in peripheral nerve block, a large number of cases reported in several countries support the use of peripheral nerve block instead of GEA.^[11,17,18]^ Although reports have described the effects of peripheral nerve block in patients undergoing major LEA, such as above- or below-knee amputation, no studies have investigated these effects in patients undergoing minor LEA, such as distal foot amputation.^[16,19,20]^

Therefore, in this retrospective observational study, we compared the effects of 2 different types of anesthetic modalities, remifentanil-based GEA and popliteal nerve block (PNB), on postoperative pain and hemodynamic stability in diabetic patients undergoing distal foot amputation. Furthermore, the effects of these modalities on postoperative pulmonary complications and 6-month mortality were evaluated.

## 2 Methods

**Figure 1:**
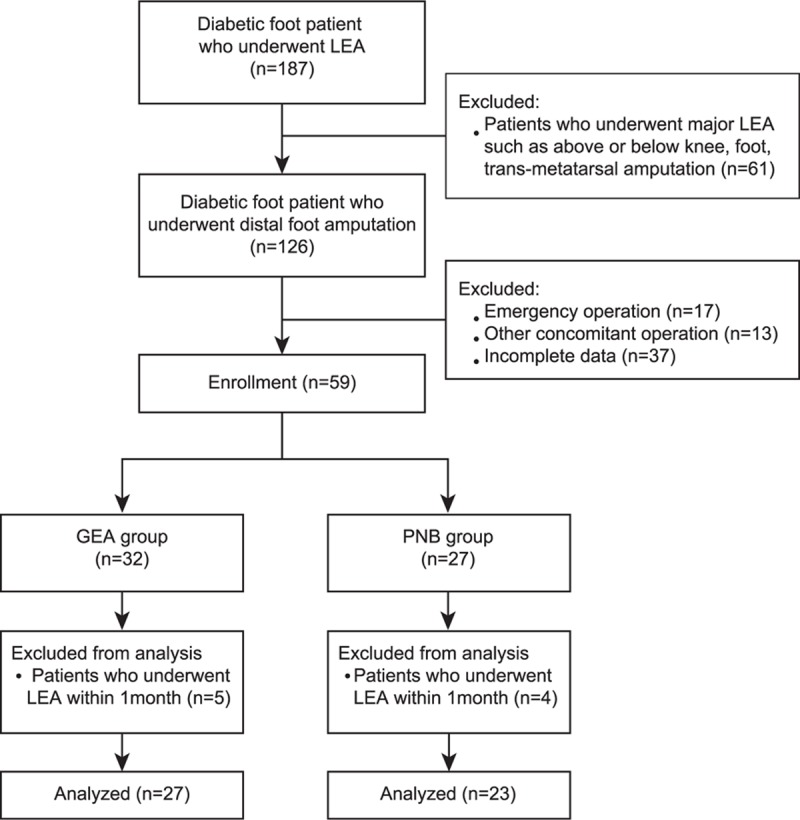
Flow chart of the study. GEA = general anesthesia, LEA = lower extremity amputation, PNB = popliteal nerve block.

This was a retrospective review of patients with a diabetic foot who underwent distal foot amputation between January 2012 and May 2014 at Severance Hospital, Seoul, Republic of Korea. Clinical data were analyzed after receiving approval from the Institutional Review Board and Hospital Research Ethics Committee of Severance Hospital (Yonsei University Health System, Seoul, Republic of Korea; IRB protocol no. 4-2016-0233). The requirement for informed patient consent was waived; however, patient records were anonymized and deidentified prior to analysis. For the purpose of this study, distal foot amputation was defined as a minor foot amputation, such as toe amputation or debridement, or partial foot amputation (limited to a surgical level distal to only the metatarsophalangeal joint level; surgeries involving more proximal levels were excluded). Patients with the following conditions were excluded: emergency cases and patients who underwent major LEA or other concomitant surgeries, had incomplete data, and underwent any LEA within 1 month (Fig. 1). Patients received remifentanil-based GEA (GEA group, n = 32) or PNB (PNB group, n = 27). None of the patients were administered premedication. After applying standard equipment to monitor the mean blood pressure (MBP), electrocardiography, and oxygen saturation, different types of anesthesia were induced as described in the following. All surgeries were performed without applying a pneumatic tourniquet.

In the GEA group, propofol (1–1.5 mg/kg), rocuronium (0.6 mg/kg), and remifentanil infusion (0.05–0.1 μg/kg/min) were used as the standard regimen. To maintain an end-tidal CO_2_ of 35 to 42 mm Hg, mechanical ventilation of 40% oxygen in air was performed at a respiratory rate of 10 to 20 breaths/min by applying a tidal volume of 6 to 8 mg/kg of the ideal body weight, a positive end-expiratory pressure of 5 cm H_2_O, and a 1:2 inspiratory-to-expiratory time ratio. All patients underwent traditional inhalation anesthesia with sevoflurane (0.6–1.0 age-adjusted minimal alveolar concentration) and remifentanil (0.03–0.5 μg/kg/min) while maintaining a target range of 40 to 60 on a bispectral index monitor (Aspect A-2000; Aspect Medical System Inc, Newton, MA).

In the PNB group, popliteal sciatic nerve block was performed by a single experienced anesthesiologist. Patients were placed in the lateral position and prepared by placing sterile draping over the affected popliteal fossa. A SonoSite M-Turbo ultrasound unit (SonoSite Inc, Bothell, WA) with a 7.5-MHz linear probe and a 50-mm, 22-gauge block needle was used for every patient. The skin and subcutaneous tissues at the site of needle entry were anesthetized with 1% lidocaine. A standard local anesthetic solution of equal parts of 2% plain lidocaine and 0.75% ropivacaine was used at a total volume of 25 mL. The sciatic nerve was identified in the transverse plane, and the block was performed at the site of sciatic nerve bifurcation.^[21]^ After completing the procedure, the sensory and motor blocks were assessed. Sensory function was assessed as sensation to pinprick to the plantar and dorsum surface of the foot. Motor function was assessed by ankle movement power. Surgery was initiated after verification of complete sensory and motor blocks. Anxious or agitated patients received a small dose of midazolam (0.02 mg/kg) intravenously.

The following preoperative data were assessed: demographic data and preexisting conditions such as hypertension, chronic kidney disease, cerebrovascular accident, or coronary artery occlusive disease. Preoperative laboratory data, including hematocrit level, platelet counts, prothrombin time, activated prothrombin time, and glycated hemoglobin level, were recorded. Moreover, the use of preoperative medications with anticoagulant effects was recorded. The assessed intraoperative data were the duration of surgery, MBP, and heart rate (HR) during surgery. The intraoperative fluid amount, blood loss volume, ephedrine dose, and transfusion requirement were also assessed. Postoperative pain scores were evaluated using the numeric rating scale (NRS; 0 = no pain at all and 10 = worst pain imaginable).^[18]^ Patients were informed to ask for additional analgesics when their pain score reached or exceeded 4. If the pain score exceeded 4 or patients requested additional analgesics, intramuscular pethidine was administered in 12.5-mg increments. The first rescue analgesic requirement during the first 24 hours after surgery was also evaluated. The following postoperative data were assessed: number of patients admitted to the intensive care unit (ICU), duration of postoperative hospital stay, and postoperative complications, including pneumonia, development of an acute kidney injury, deterioration of mental status, heart failure, and reoperation within 1 month. Furthermore, we assessed the mortality of all patients until 6 months after surgery.

### 2.1 Statistical analysis

All statistical analyses were performed using IBM SPSS Statistics 20.0 (SPSS Inc, Chicago, IL), SAS version 9.2 (SAS Institute, Cary, NC), and R version 3.0.3 (R Project for Statistical Computing, Vienna, Austria). Continuous variables were presented as means ± standard deviation, and dichotomous variables were presented as number of patients (percentage). The analysis of parametric data was performed using the independent *t* test, and dichotomous variables were analyzed using the chi-square test. A linear mixed model was used to analyze repeatedly measured variables such as NRS scores, MBP, and HR. A comparison of differences between the groups over time was performed using a group by time interaction. Post hoc analyses with Bonferroni correction for multiple comparisons were performed when statistical significance was observed in the repeated measures analysis. Furthermore, first postoperative rescue analgesic requests were analyzed using the Kaplan–Meier survival method and log-rank test. A *P* value of <0.05 was considered statistically significant.

## 3 Results

Data from 187 consecutive patients who underwent LEA at Severance Hospital from January 2012 to May 2014 were obtained from electronic medical records. After removing cases that involved major LEAs, such as above-knee or below-knee amputation, a total of 126 patients were evaluated for eligibility. Sixty-seven patients were excluded because of emergency surgery, other concomitant surgeries, or incomplete data, leaving 59 patients who were reviewed. After a total of 9 patients who underwent LEA within 1 month were excluded from both groups, the remaining 50 patients were subjected to the final analysis (Fig. 1). None of the patients experienced an incomplete block or developed PNB-related complications such as hematoma or infection. The 2 groups were comparable with respect to demographic features, except for the number of the patients taking preoperative clopidogrel medication (Table 1).

**Table 1:**
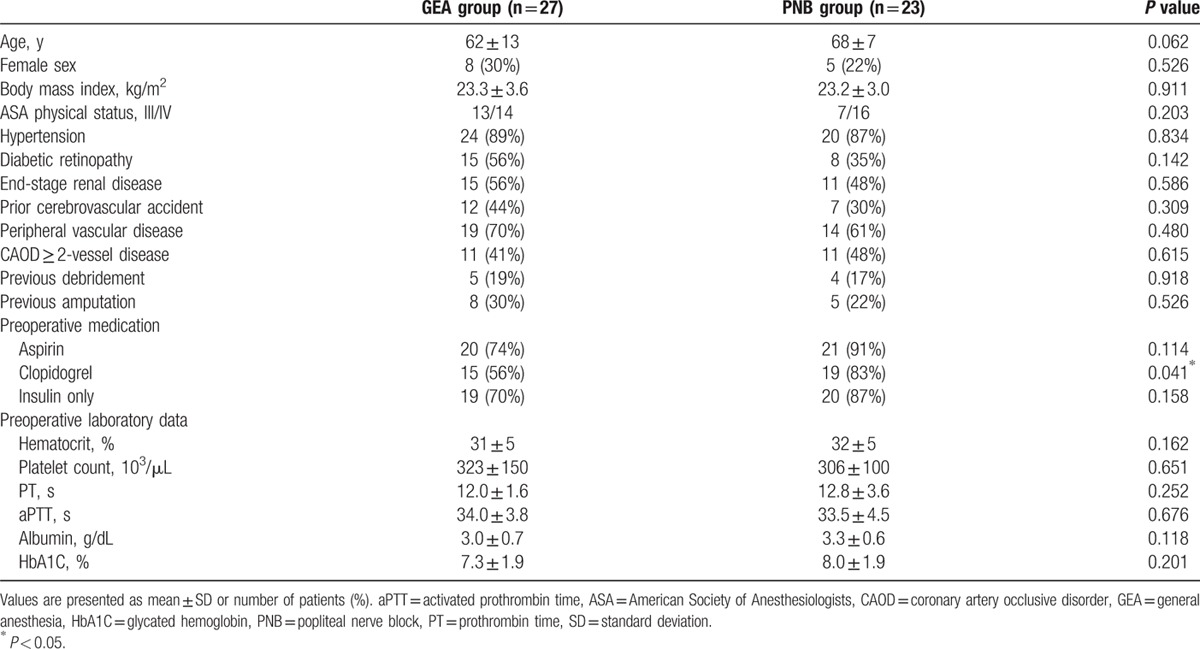
Patients’ characteristics.

**Figure 2:**
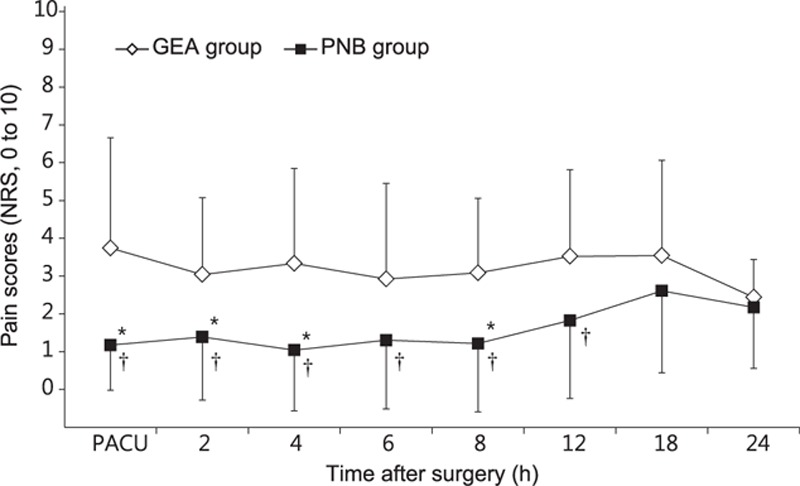
Postoperative pain scores during the first 24 hours after surgery. Values are presented as means ± SD. (∗) *P* < 0.05, versus the GEA group (Bonferroni corrected); (†) *P* < 0.05, versus the GEA group. GEA = general anesthesia, NRS = numeric rating scale, PACU = postanesthetic care unit, PNB = popliteal nerve block, SD = standard deviation.

**Figure 3:**
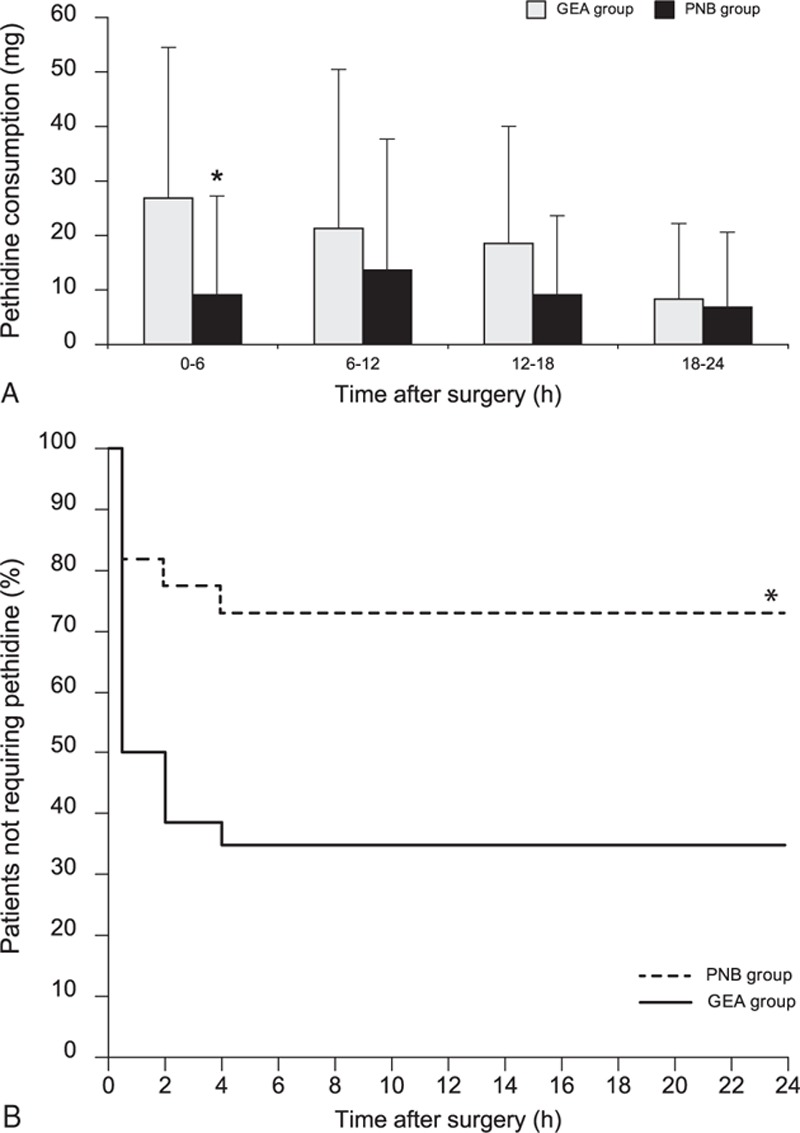
Consumed pethidine doses during the first 24 hours after surgery (A) and Kaplan–Meier analysis of the time to the first required rescue analgesic (B). Values are presented as means ± SD or the number of patients (%). (∗) *P* < 0.05, versus the GEA group. GEA = general anesthesia, NB = popliteal nerve block, SD = standard deviation.

Significant differences in NRS scores were observed between the 2 groups in the linear mixed model analysis (*P*_Group×Time_ = 0.044). Even after a post hoc analysis with the Bonferroni correction, the NRS scores were significantly lower in the PNB group than in the GEA group on arrival in the postanesthetic care unit and at 2, 4, and 8 hours after surgery (Fig. 2). The highest pain score during the 24-hour period after surgery was significantly lower in the PNB group than in the GEA group (3.3 ± 2.1 vs 5.4 ± 2.7; *P* = 0.005). In addition, patients in the PNB group required less pethidine than did those in the GEA group during the first 6 hours after surgery (27 ± 28 vs 9 ± 18 mg; *P* = 0.013) (Fig. 3A); as shown in Fig. 3B, the Kaplan–Meier curve indicates that the proportion of the patients who did not require rescue pethidine during the first 24 hours after surgery was higher in the PNB group than in the GEA group (*P* = 0.008).

**Figure 4:**
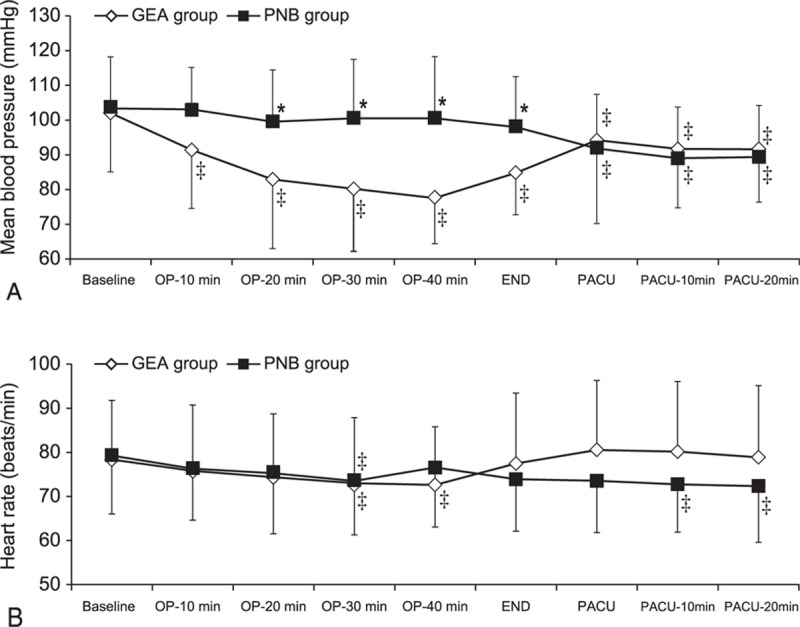
The mean blood pressure (A) and heart rate (B) during the perioperative period. Values are presented as means ± SD. (∗) *P* < 0.05, versus the GEA group (Bonferroni corrected); (‡) *P* < 0.05, versus the baseline value for each group. GEA = general anesthesia, OP = operation, PACU = postanesthetic care unit, PNB = popliteal nerve block, SD = standard deviation.

The intraoperative variables are listed in Table 2. Among these variables, the total fluid intake and administered ephedrine dose differed significantly between the 2 groups (Table 2). Although a higher dose of ephedrine was administered in the GEA group, compared to that in the PNB group (*P* < 0.001), MBPs in the GEA group were significantly lower than those in the PNB group from 20 minutes after the initiation of surgery to the end of surgery (Bonferroni-corrected *P* < 0.01). When compared with the baseline MBP values, MBP was significantly reduced across all time points in the GEA group; in contrast, the PNB group exhibited reductions in MBP only at the time points measured in the postanesthetic care unit (Fig. 4A). No significant difference in HR was observed between the groups (Fig. 4B).

**Table 2:**
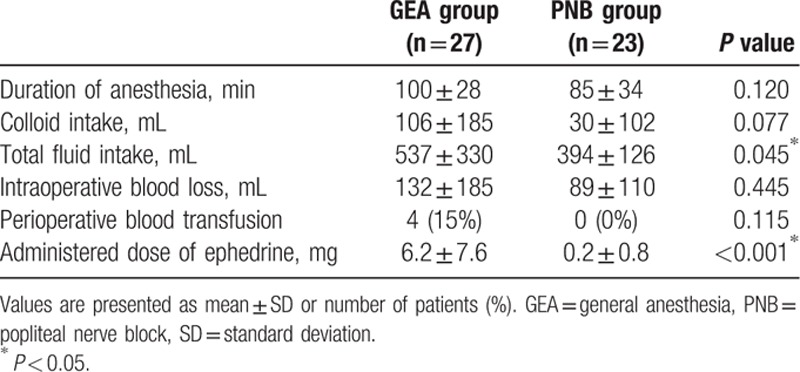
Intraoperative variables.

The postoperative outcomes are presented in Table 3. The duration of hospital stay was comparatively shorter in the PNB group, although this difference was not significant. The rate of pneumonia development after surgery was significantly higher in the GEA group than in the PNB group (*P* = 0.030). After surgery, some of the patients from both groups received care in the ICU. Notably, significantly more patients in the GEA group received care in the ICU, compared with patients in the PNB group (*P* = 0.038). Although 3 and 1 cases of mortality were observed within 6 months of surgery in the GEA and PNB groups, respectively, this difference was not statistically significant.

**Table 3:**
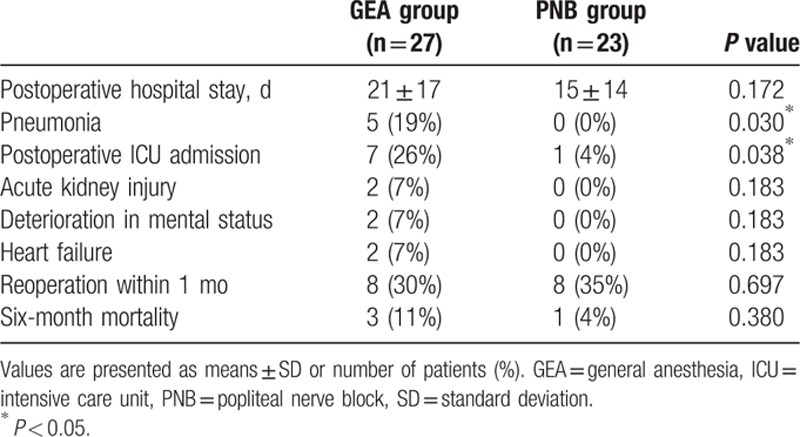
Postoperative outcomes.

## 4 Discussion

This was the first study to retrospectively review and compare anesthetic modalities with respect to postoperative pain and hemodynamic stability in patients with a diabetic foot undergoing minor LEA. We found that a PNB provided a sufficient postoperative analgesic effect while maintaining hemodynamic stability when compared with GEA. Furthermore, a larger proportion of patients who received GEA, compared with patients who received PNB, suffered from pneumonia during the postoperative period and received care in the ICU after surgery.

Patients with persistent DM are of particular concern to anesthesiologists because of the high risk of multiorgan failure, which is associated with many neuropathic, nephropathic, and microangiopathic diseases.^[15]^ For these patients, it is well recognized that peripheral nerve block is correlated with improved postoperative analgesia, increased functional recovery rate, and alleviated hemodynamic instability during the perioperative period.^[22,23]^ The rate of foot amputations in patients with DM may be approximately 75%, and the incidence of reamputation among patients with diabetes has been well recognized.^[9]^ Although, in diabetic patients with foot problems, most surgical treatments are initiated at the distal level of the affected foot,^[9]^ none of the previous reports regarding the effects of peripheral nerve block have evaluated in the minor levels of surgery. Therefore, the present study focused on the effects of anesthetic modalities on postoperative pain and hemodynamic stability in diabetic patients undergoing amputation at the distal level of the affected foot.

This study demonstrated that PNB has several advantages over GEA during the intraoperative and immediate postoperative periods, which were consistent with previous reports.^[22,23]^ During surgery, PNB clearly provided better hemodynamic stability, compared to GEA. Patients in the GEA group were more frequently treated with ephedrine because of hypotension events and received larger volumes of fluids compared to those in the PNB group. Notably, the MBP during surgery was significantly lower in the GEA group than in the PNB group; this might be attributable to the potential of GEA, which involves anesthesia and mechanical ventilation, to aggravate hypotension in patients with preexisting dehydration. This issue may be very decisive among diabetic patients with a preoperative poor physiological status.

We observed significantly lower NRS scores in the PNB group than in the remifentanil-based GEA group, which were consistent with previous results.^[22,23]^ Moreover, patients in the GEA group required significantly higher doses of rescue analgesics than did those in the PNB group during the first 6 hours after surgery. Furthermore, the proportion of the patients who did not require rescue pethidine during the first 24 hours after surgery was significantly higher in the PNB group than in the GEA group. This might be attributable not only to the nature of the nerve block in the PNB group but also to the short-acting opioid effects of remifentanil, which was used in the GEA group. In addition, many reports have described the potential for remifentanil-induced hyperalgesia, which might affect postoperative pain intensity in the GEA group.^[24–26]^

Although pain control has been considered an important component of postoperative management, in high-risk patient populations—such as those with severe comorbidities—pain control is known to be a crucial element in the control of postoperative complications.^[27,28]^ All patients in both groups had an American Society of Anesthesiologists physical status score higher than III; 14 (52%) in the GEA group and 16 (70%) in the PNB group had an American Society of Anaesthesiology physical status score of IV. In actual clinical practice, many anesthesiologists might hesitate to administer anesthesia to critically ill patients undergoing LEA, especially those receiving anticoagulation therapy. Actually, inconsistencies and controversies still remain regarding the mortality benefits of anesthetic modalities in such patients. Similarly, we could not find any significant differences in the mortality rate. However, in the present study, a significantly higher number of patients in the GEA group suffered from pulmonary complications after surgery and required ICU care after surgery, compared to patients in the PNB group. The anesthetic management of such patients might introduce greater concerns and challenges with respect to the requirement for ICU care and related expenditures, and even if only a single minor surgery is performed (i.e., only debridement or distal amputation), reamputation of more proximal levels will happen in about 30% to 40% of cases.^[9]^ Therefore, when compared with repeated GEA, PNB might be a valuable anesthetic technique with regard to patient safety and economy, especially for high-risk diabetic patients with comorbid diseases who are undergoing distal foot surgery.^[29,30]^

This study has several limitations. Owing to an inherent limitation of this retrospective study, we excluded cases with incomplete information from our analysis, which likely caused selection bias. In addition, this study was conducted in a single clinical setting and involved a small sample size with a homogeneous racial background, which not only might limit the ability to detect potentially significant associations but would also render the findings inapplicable to other clinical settings. Furthermore, this study was observational in nature; the attending anesthesiologists selected the anesthetic techniques, and their decisions might have been influenced by their judgments and preconceived notions. Finally, this study did not demonstrate a significant mortality benefit of PNB among diabetic patients who underwent distal foot amputation, likely because of an insufficiently powerful sample size. Therefore, additional investigations, including larger, more controlled studies, are needed to evaluate the influences of anesthetic techniques on long-term morbidity and mortality in patients with a diabetic foot who may be at a high risk of repeated surgery.

In conclusion, this study demonstrated that compared to remifentanil-based GEA, PNB might be a favorable option for diabetic patients undergoing distal foot amputation, despite the lack of significant mortality benefits, as PNB is associated with improved postoperative analgesia, hemodynamic stability, and a low incidence of pulmonary complications during immediate postoperative period, especially in high-risk patients.

## Acknowledgments

The authors thank the biostatisticians of the Department of Research Affairs for their statistical comments and analysis as well as Dong-Su Jang, MFA, medical illustrator, Medical Research Support Section, Yonsei University College of Medicine, for his help with the figures.
